# GLP-1 Mediates Regulation of Colonic ACE2 Expression by the Bile Acid Receptor GPBAR1 in Inflammation

**DOI:** 10.3390/cells11071187

**Published:** 2022-04-01

**Authors:** Michele Biagioli, Silvia Marchianò, Rosalinda Roselli, Cristina Di Giorgio, Rachele Bellini, Martina Bordoni, Eleonora Distrutti, Bruno Catalanotti, Angela Zampella, Luigina Graziosi, Annibale Donini, Stefano Fiorucci

**Affiliations:** 1 Dipartimento di Medicina e Chirurgia, Università di Perugia, 06132 Perugia, Italy; michele.biagioli@unipg.it (M.B.); silvia4as@hotmail.it (S.M.); cristi.digiorgio@gmail.com (C.D.G.); rachelebellini92@gmail.com (R.B.); mbordoni92@gmail.com (M.B.); annibale.donini@unipg.it (A.D.); 2 Department of Pharmacy, University of Naples Federico II, 80138 Naples, Italy; rosellirosalinda@yahoo.it (R.R.); bruno.catalanotti@unina.it (B.C.); azampell@unina.it (A.Z.); 3 SC di Gastroenterologia ed Epatologia, Azienda Ospedaliera di Perugia, 06132 Perugia, Italy; eleonoradistrutti@katamail.com (E.D.); luiginagraziosi@yahoo.it (L.G.)

**Keywords:** ACE2, intestinal inflammation, SARS-CoV-2, bile acids, GPBAR1, GLP-1

## Abstract

Background & Aims: ACE2, a carboxypeptidase that generates Ang-(1-7) from Ang II, is highly expressed in the lung, small intestine and colon. GPBAR1, is a G protein bile acid receptor that promotes the release of the insulinotropic factor glucagon-like peptide (GLP)-1 and attenuates intestinal inflammation. Methods: We investigated the expression of ACE2, GLP-1 and GPBAR1 in two cohorts of Crohn’s disease (CD) patients and three mouse models of colitis and Gpbar1^−/−^ mice. Activation of GPBAR1 in these models and in vitro was achieved by BAR501, a selective GPBAR1 agonist. Results: In IBD patients, ACE2 mRNA expression was regulated in a site-specific manner in response to inflammation. While expression of ileal ACE2 mRNA was reduced, the colon expression was induced. Colon expression of ACE2 mRNA in IBD correlated with expression of TNF-α and GPBAR1. A positive correlation occurred between GCG and GPBAR1 in human samples and animal models of colitis. In these models, ACE2 mRNA expression was further upregulated by GPABR1 agonism and reversed by exendin-3, a GLP-1 receptor antagonist. In in vitro studies, liraglutide, a GLP-1 analogue, increased the expression of ACE2 in colon epithelial cells/macrophages co-cultures. Conclusions: ACE2 mRNA expression in the colon of IBD patients and rodent models of colitis is regulated in a TNF-α- and GLP-1-dependent manner. We have identified a GPBAR1/GLP-1 mechanism as a positive modulator of ACE2.

## 1. Introduction

The angiotensin-converting enzyme (ACE) is a dipeptidyl-peptidase exerting a critical role in the renin–angiotensin system (RAS). ACE exists in two main isoforms (ACE and ACE2) sharing approximately 42% homology in the N-terminal catalytic domain [1], but with different substrate specificity: Thus, while ACE principally functions as a peptidyl dipeptidase removing the C-terminal dipeptide from Angiotensin (Ang) I to form Ang II, ACE2 functions exclusively as a carboxypeptidase removing a single amino acid from the C-terminal Ang II to generate Ang-(1-7), or with much less efficiency, Ang-(1-9) from Ang I. ACE and ACE2 regulate the generation of a family of vasoactive peptides that act through three distinct receptors, AT_1_ and AT_2_ and MAS (MAS1 protooncogene G protein coupled receptor (GPCR) to regulate vasoconstriction and vasodilatation in the rennin-angiotensin system (RAS). Despite structural similarities, ACE2 activity is not inhibited by classical ACE inhibitors [1]. In the normal lung, ACE2 mRNA is mainly expressed by type II alveolar epithelial cells and endothelial cells [2,3], but the level of expression increases in response to inflammation or exposure to interferon (IFN)-γ [4]. In addition, ACE2 has been identified as the receptor for the spike protein of virus SARS-CoV-2 [5].

In addition to the lung, ACE2 mRNA and protein, are highly expressed in the gastrointestinal tract, with the higher expression detected in epithelial cells of the ileum and the colon [6]. In the intestinal epithelial cells, ACE2 associates with the neutral amino acid transporter B^0^AT1 (*SLC6A19*) and is required for expression of this transporter on the luminal surface of intestinal epithelial cells. ACE2 expression in the intestine undergoes regulation in response to a variety of factors including intestinal microbiota and inflammation [7] and transplantation of intestinal microbiota from Ace2 mutant mice into germ-free wild-type recipients increases the propensity of these mice to develop severe colitis [8]. Together, these findings indicate that ACE2 exerts a role as a regulator of dietary amino acid homeostasis, innate immunity and gut microbial ecology [7,8]. Nevertheless, mechanisms that regulate ACE2 expression in the intestines remain poorly defined.

Crohn’s disease (CD) and ulcerative colitis (UC) are two highly prevalent inflammatory disorders of the intestine (IBD) that develop in genetically susceptible individuals in response to an altered immune response to the intestinal microbiota, and are treated by conventional anti-inflammatory and immunosuppressive drugs and biologic agents [9,10,11]. Since ACE2 contributes to regulation of intestinal microbiota composition and intestinal immunity, understanding of mechanisms of regulation might be of therapeutic relevance in IBD [12,13]. However, despite the fact that available information suggests that ACE2 expression is differentially regulated in response to inflammation in the ileum and colon of IBD patients, only a limited number of regulatory mechanisms have been identified [14].

Bile acids are a family of atypical steroids generated from cholesterol at the interface of host and microbial metabolism. Indeed, while the two main human primary bile acids, chenodeoxycholic acid and cholic acid (CDCA and CA), are generated in the liver from cholesterol breakdown, secondary bile acids, deoxycholic and lithocholic (DCA and LCA) are products of bio-transformations operated by the intestinal microbiota [15,16,17]. As such, secondary bile acids are part of chemical communications that connect the intestinal microbiota with the host [18]. In addition to their role in nutrient absorption, and similarly to other steroids, bile acids activate a family of cell membrane and nuclear receptors [16,17]. Secondary bile acids have been identified as the physiological ligands to the cell membrane receptor known as the G protein bile acid receptor (GPBAR)1, also known as TGR5 [19,20]. GPBAR1 is robustly expressed by intestinal epithelial cells, ileal and colon, intestinal endocrine cells (L cells), endothelial cells and neurons, along with immune cells of myeloid origin, exerting a number of genomic and non-genomic effects [21,22,23,24]. In the small intestine, GPBAR1 activation promotes the release of the insulinotropic factor glucagon like peptide (GLP)-1 that promotes insulin release from pancreatic beta cells [25,26]. We have previously shown that GPBAR1 gene ablation results in a phenotype that is prone to develop intestinal inflammation [27] and that GPBAR1 agonists prevent intestinal inflammation in variety of rodent models of IBD [28]. Previous studies have suggested that GLP-1 might regulate ACE2 expression in the heart [29], suggesting a potential interaction of GLP-1 agonists with ACE2. Whether this interaction operates in response to activation of GPBAR1 in the small intestine or colon is unknown.

Here, by using genetic and pharmacological approaches, we report that colonic expression of ACE2 is induced in response to intestinal inflammation in humans and rodent models of colitis in a GPBAR1-dependent manner and that the GPBAR1–GLP-1 axis mediates this regulation, overcoming inflammation.

## 2. Material and Methods

### 2.1. Human Tissue Sample

Biological samples of healthy and inflamed colon and ileum mucosa were collected from 7 Crohn’s disease (CD) patients, undergoing ileal resection for untreatable disease at the Perugia’s Santa Maria della Misericordia hospital. Permission to collect post-surgical samples was granted to Prof. Fiorucci by the University of Perugia’s bioethics committee, originally in 2014 and on the 1 December 2020; Permit FIO0003, n. 120687. Informed written consent was obtained from each patient before surgery.

### 2.2. GEO Data Sets

The GSE111889 series includes gene expression profiles (RNA-seq analysis, Illumina HiSeq 2000) of biopsy samples retrieved from CD, UC and non-IBD patients enrolled in the Human Microbiome Project, part 2 (HMP2) project [30].

### 2.3. Animals

Balb/c mice were purchased from Charles River. GPBAR1 null mice (generated directly into C57BL/6NCrl background), and congenic littermates on C57BL/6NCrl mice were originally donated by Dr. Galya Vassileva (Schering-Plough Research Institute, Kenilworth). The colonies were maintained in the animal facility of the University of Perugia. Mice were housed under controlled temperatures (22 °C) and photoperiods (12:12-h light/dark cycle), allowed unrestricted access to standard mouse chow and tap water and allowed to acclimate to these conditions for at least 5 days before inclusion in an experiment. The study was conducted in compliance with Italian law and the protocol was approved by an ethical committee of the University of Perugia and by a national committee of the Ministry of Health (permission n. 1126/2016-PR and n. 583/2017-PR). The health and body conditions of the animals were monitored daily by the Veterinarian in the animal facility. The study protocol caused minor suffering, however, animals that lost more than 25% of the initial body weight were euthanized.

### 2.4. Mouse Models of Colitis

We used three mouse models of chemically induced colitis to best simulate the broad spectrum of human IBDs. Colitis caused by trinitrobenzenesulfonic acid (TNBS) was induced in Balb/c mice as in Gpbar1^+/+^ and Gpbar1^−/−^ mice. In brief, mice were fasted for 1 day (day called −1). The day after (day 0) mice were anesthetized, and a 3.5 F catheter inserted into the colon such that the tip was 4 cm proximal to the anus. To induce colitis, 1 mg of TNBS (Sigma Chemical Co., St Louis, MO, USA) in 50% ethanol was administered via catheter into the lumen using a 1 mL syringe (injection volume of 100 μL); control mice received 50% ethanol alone. When prompted by the experimental design the BAR501 (30 mg/Kg) was administered by gavage (o.s.) alone or in combination with exendin-3 (Ex-3) (30 μg/mice) by i.p.

An oxazolone mouse model of colitis was induced in Balb/c mice. In brief, mice were anesthetized and a pre-sensitization step was performed: 150 µL of the 3% oxazolone (Sigma) in 4:1 acetone/olive oil solution was applied on a 1.5 × 1.5–cm area of shaved skin on the back of the mouse. Seven days later, mice were anesthetized and 150 µL of 1% oxazolone solution in 50% ethanol was administered per rectum. Mice were sacrificed four days after treatment and clinical signs of colitis were scored. When prompted by the experimental design the BAR501 (30 mg/Kg) was administered by gavage (o.s.).

Colitis caused by DSS was induced in Balb/c mice by administering, for 8 consecutive days, of 3% DSS (DSS: Dextran Sulfate, Sodium Salt of Affymetrix USA, molecular mass 40–50 kDa) in drinking water as described by Wirtz S. et al. [31].

Animals were monitored daily for appearance of diarrhea, loss of body weight, presence of blood in the stool and survival. At the end of the experiment, 4 days after the administration of TNBS, surviving mice were sacrificed and the colon and ileum were excised.

### 2.5. Clinical Disease Activity Index (CDAI)

The severity of colitis was measured each day for each mouse by analyzing the body weight lost, the occult blood and stool consistency. Each parameter was scored from 0 to 4 and the sum represents the Colitis Disease Activity Index (CDAI). The scoring system was as following: percent of body weight loss: none = 0; 1–5% = 1; 5–10% = 2; 10–20% = 3; >20% = 4. Stool consistency: normal = 0; soft but still formed = 1; very soft = 2; diarrhea = 3; liquid stools that stick to the anus or anal occlusion = 4. Fecal blood: none = 0; visible in the stool = 2; severe bleeding with fresh blood around the anus and very present in the stool = 4.

### 2.6. Mouse Endoscopies

For endoscopic evaluations, on the last day of the experiment, mice were anesthetized and the endoscope was rectally inserted for maximum of 2.5 cm and videos of the endoscopic procedure were recorded. Five items of endoscopic severity were scored: ‘mucosal thickening’, ‘vasculature’, ‘granularity of the mucosal surface’, ‘fibrin deposits’ and ‘stool appearance’, as proposed by Becker et al. [32].

### 2.7. Intestinal Permeability

Intestinal permeability test was performed using fluorescein isothiocyanate conjugated dextran (FITC-dextran) (Sigma-Aldrich, St. Louis, MO, USA, catalog number: FD4). FITC-dextran was dissolved in PBS at a concentration of 100 mg/mL. The evening before the test day mice were fasted overnight. In the next morning, after weighing each mouse, FITC was administered to each one at a dose of 44 mg/100 g of body weight by oral gavage. After 4 h, the mice were anesthetized by inhalation of isoflurane and 300–400 μL of blood collected from the facial vein in microtubes containing EDTA. Blood was stored in the dark. Once blood has been collected from all the mice, microtubes were processed to separate the serum. For analysis, serum was diluted with an equal volume of PBS. The concentration of FITC in serum was determined by spectrophoto fluorometry with an excitation of 485 nm and an emission wavelength of 528 nm using as a standard serially diluted FITC-dextran (0, 125, 250, 500, 1000, 2000, 4000, 6000, 8000 ng/mL). Serum from mice not administered with FITC-dextran was used to determine the background.

### 2.8. Determination of Blood Glucose Levels

When required by the experimental set, before the sacrifice of the mice, the blood glucose concentrations were measured using a portable glucose meter (Accu-Check Go., Roche, Basel, Switzerland).

### 2.9. Histopathology

Colon samples (2–3 cm up the anus) were first fixed in 10% Formalin (Bio-Optica Milano S.p.A. Milan, IT, Italy), embedded in Paraffin (Bio-Optica Milano S.p.A. Milan, IT, Italy), cut into 5-μm-thick sections and then stained with Hematoxylin/Eosin (H&E) (Bio-Optica Milano S.p.A. Milan, IT, Italy) for histopathological analysis.

### 2.10. Immunohistochemistry

Immunohistochemistry was performed on paraffin embedded human colon. In brief, Ag retrieval was achieved by incubation of the slides for 90 min in the hot (95 °C) sodium citrate buffer (pH 6.0) and 30 min of cooling at room temperature. Immunostaining technique was carried out using the commercial kit Elabscience^®^2-step plus Poly-HRP Anti Rabbit/Mouse IgG Detection System (with DAB Solution) (Houston, TX, USA) ACE2 Recombinant Rabbit Monoclonal Antibody (SN0754) Invitrogen, Thermofisher scientific (Waltham, MA, USA) was incubated overnight at 4 °C. Subsequently, sections were incubated with Polyperoxidase-anti-Mouse/Rabbit IgG and then with DAB Working Solution, both supplied by the kit. Slides were counterstained with hematoxylin, dehydrated through ethanol and xylene, and coverslipped using a xylene-based mounting medium.

Immunocytochemistry was instead performed on HT29 stimulated with IL-1β, TNF-αand BAR501.

Cells were plated on slides using cytospin. The spots obtained were fixed in 4% formalin for 20 min and then subjected to the same procedure of immunostaining with the commercial kit Elabscience^®^2-step plus Poly-HRP Anti Rabbit/Mouse IgG Detection System (with DAB Solution) (Houston, TX, USA). After incubation with ACE2 primary antibody and secondary antibody supplied by the kit, cells were counterstained with hematoxylin and then observed under microscope with magnification 100×.

### 2.11. Cell Culture

HT-29 cells, a human colorectal adenocarcinoma cell line was grown at 37 °C in D-MEM containing 10% FBS, 1% l-glutamine, and 1% penicillin/streptomycin. Cells were regularly passaged to maintain exponential growth. U937 cells, a monocyte cell line were cultured at 37 °C in RPMI supplemented with 10% FBS, 1% glutamine, and 1% penicillin/streptomycin.

Approximately 2 × 106 HT-29 cells were seeded and activated with TNF-α (100 ng/mL, SinoBiological) + IL1b (25 ng/mL) for 24 h alone or in combination with BAR501 (2.5, 5, 10, 20 µM) and liraglutide (10 nM).

U937 and HT29 cells were used to perform a co-culture experiment. Briefly, HT29 cells (1.5 × 106 cells/well) were seeded in 6-well plates and cultured for 24 h. After 10 h of starvation U937 (106 cells/well) were added to each well and both cell lines were stimulated with LPS (100 ng/mL) alone or in combination with BAR501 (10 µM), liraglutide (10 nM) and liraglutide + BAR501. After 24 h both cell types were separately collected to perform RNA extraction.

### 2.12. RNA Extraction

RNA was extracted from human biopsies, mouse colon and ileum and HT-29 cells using TRIzol reagent (Invitrogen) and Direct-zol™ RNA MiniPrep w/ Zymo-Spin™ IIC Columns (Zymo Research, Irvine, CA, USA). RNA samples extracted from DSS-treated colon tissue were subjected to a LiCl precipitation according the procedure of Viennois E. et al. [33]. Briefly, 0.1 vol of 8 M LiCl (Sigma-Aldrich, St. Louis, MO, USA) in RNase-free water was added to the RNA elution and the samples were precipitated at 4 °C for 2 h. Samples were then centrifuged at 14,000 × *g* for 30 min at 4 °C and the supernatant carefully decanted. The resulting pellet was gently resuspended in RNase-free water and the LiCl precipitation was repeated. As LiCl is a known PCR inhibitor, the samples were precipitated once more for 30 min at −20 °C in 0.1 vol of 3 M sodium acetate (NaOAc), pH 5.2 and 2 volumes of 100% ethanol. Samples were centrifuged at 14,000 × *g* for 30 min and 4 °C, and the supernatant decanted. The resulting pellet was washed twice with 70% ethanol to remove excess salt and resuspended in RNase-free water.

### 2.13. Reverse Transcription of mRNA and Real-Time PCR

After purification from genomic DNA using DNase I (Thermo Fisher Scientific, Waltham, MA, USA), 1 μg of RNA from each sample was reverse transcribed using a FastGene Scriptase Basic Kit (Nippon Genetics Europe) in a 20-μL reaction volume; 50 ng of cDNA was amplified in a 20-μL solution containing 200 nM of each primer and 10 μL of SYBR Select Master Mix (Thermo Fisher Scientific, Waltham, MA, USA). All reactions were performed in triplicate using the following thermal cycling conditions: 3 min at 95 °C, followed by 40 cycles of 95 °C for 15 s, 56 °C for 20 s and 72 °C for 30 s, using a StepOnePlus system (Applied Biosystems, Foster City, CA, USA). The relative mRNA expression was calculated according to the ΔCt method. Primers were designed using the software PRIMER3 (http://frodo.wi.mit.edu/primer3/, accessed on 1 January 2021), using data published in the NCBI database. The primer used were as following (forward and reverse): hGapdh (for CAGCCTCAAGATCATCAGCA; rev GGTCATGAGTCCTTCCACGA), hGusB (for GAGCCTGCGTCCCACCTA; rev TGCTCACAAAGGTCACAGG), hACE2 (for GGACCCAGGAAATGTTCAGA; rev CGTCCATTGTCACCTTTGTG), hTNF-α (for AGCCCATGTTGTAGCAAACC; rev TGAGGTACAGGCCCTCTGAT), hIL-6 (for AGTGAGGAACAAGCCAGAGC; rev CAGGGGTGGTTATTGCATCT), hTmprss2 (for CACTGCGTGGAAAAACCTCT; rev TGGTATCCGGCTCCATAGAA); hGpbar1 (for ACTGTTGTCCCTCCTCTCCCT; rev GACACTGCTTTGGCTGCTTG); hIl1β (for GTGGCAATGAGGATGACTTG; rev GGAGATTCGTAGCTGGATGC), hCcl2 (for TAGCAGCCACCTTCATTCCC; rev CTGCACTGAGATCTTCCTATTGG), hIcam1 (for TGATGGGCAGTCAACAGCTA; rev ACCTGGCAGCGTAGGGTAAG), mGapdh (for CTGAGTATGTCGTGGAGTCTAC; rev GTTGGTGGTGCAGGATGCATTG), mIfn-γ (for GCTTTGCAGCTCTTCCTCAT; rev ATCCTTTTGCCAGT), mTnf-α (for CCACCACGCTCTTCTGTCTA; rev AGGGTCTGGGCCATAGAACT), mIl-6 (for CTTCACAAGTCGGAGGCTTA; rev TTCTGCAAGTGCATCATCGT), mIl-1β (for GCTGAAAGCTCTCCACCTCA; rev AGGCCACAGGTATTTTGTCG), mAce2 (for AGATGGCCGGAAAGTTGTCT; rev GGGCTGTCAAGAAGTTGTCC), mMas (for CTGCTGACAGCCATCAGTGT; rev ACAGAAGGGCACAGACGAAT), mTmprss2 (for GCAAGCCTCAACATCTGTCA; rev GAGGGCTAAACACAGCGATT), mTGFβ (for TAATGGTGGACCGCAACAAC; rev ACTGCTTCCCGAATGTCTGA).

### 2.14. Western Blot

HT-29 cells were lysed in RIPA lysis buffer containing phosphatase and protease inhibitors cocktail. Aliquots from each sample containing 50 µg of protein were separated on Novex WedgeWell 4–12% Tris-Glycine gel (Invitrogen) and transferred to nitrocellulose membrane with iBlot 2 Dry Blotting System (Invitrogen). The blots were subsequently blocked for 1 h with 5% milk powder in Tris-buffered saline (TBS)/Tween 20 at room temperature and then probed overnight (at 4 °C) with primary antibodies against GAPDH (Cell Signaling D4C6R, 1:1000), ACE2 (Invitrogen MA531395, 1:1000), NF-kb (Invitrogen 51-0500, 1:1000), p-NF-kb (Invitrogen MA5-15160, 1:1000). After overnight incubation, appropriate horseradish peroxidase-labeled secondary antibody, at a dilution of 1:10,000, was used. Positive signals were developed by Immobilon Western Chemiluminescent HRP Substrate (Merck Millipore) and quantitative densitometry analysis was performed using ImageJ Software. The degree of Nfkb phosphorylation at Ser536 was calculated as the ratio between the densitometry readings of p-NF-kb/ NF-kb.

### 2.15. Statistical Analysis

The ANOVA followed by nonparametric Mann–Whitney U test or a two-tailed unpaired Student’s *t* test were used for statistical comparisons (* *p* < 0.05) using the Prism 6.0 software (GraphPad San Diego, CA, USA).

## 3. Results

### 3.1. Regional Variation of ACE2 mRNA Expression in Intact and Inflamed Gut

**Figure 1:**
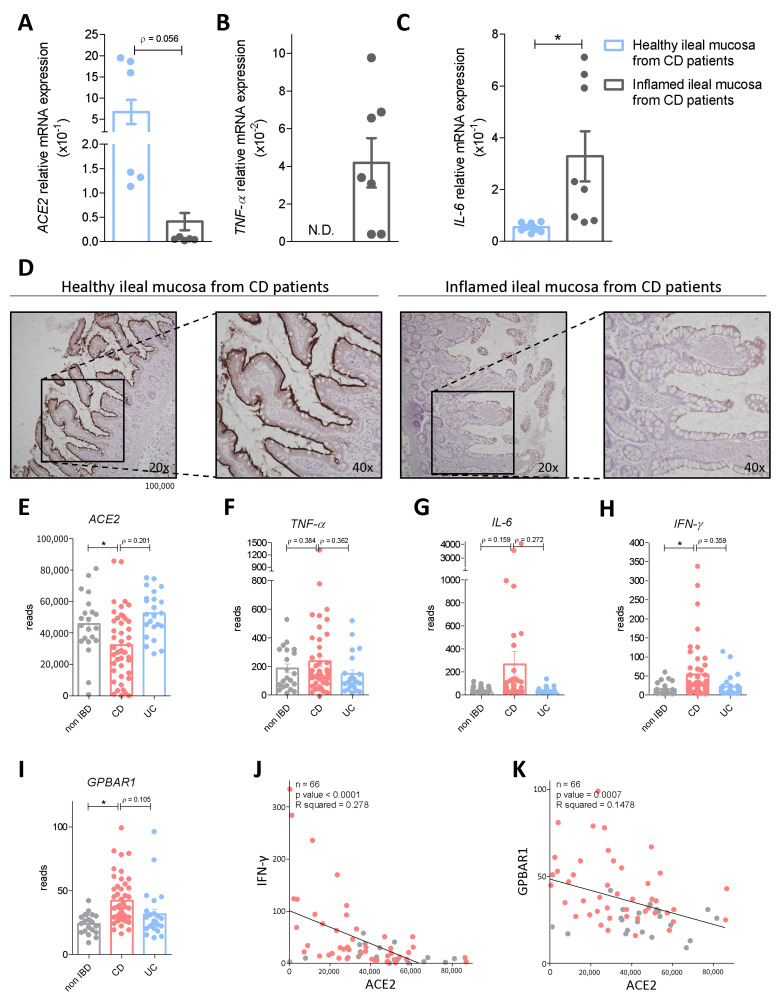
ACE2 mRNA expression varies in healthy or inflamed ileus in an opposite way to that of the colon. The relative mRNA expression of (**A**) ACE2, (**B**) TNF-α and (**C**) IL-6 in colon biopsies from healthy and inflamed ileal mucosa from Crohn’s disease (CD) patients. Data are normalized to GAPDH mRNA. Results are the mean ± SEM of 9 patients per group; * *p* < 0.05. (**D**) Immunohistochemistry analysis of ACE2 expression in non-inflamed and inflamed ileum samples obtained from CD patients (Magnification 20× and 40×). (**E–I**) RNA-seq analysis of ileal biopsy samples from HMP2 project CD, UC and non-IBD patients. Each dot represents a patient. Gene profile expression of (D) ACE2, (**E**) TNF-α, (**F**) IL-6, (G) IFN-γ (**H**) and GPBAR1. (**I–K**) Correlation graph between (**I**), INF-γ and ACE2 expression (**J**), and GPBAR1 and ACE2 (**K**). * *p* < 0.05.

**Figure 2:**
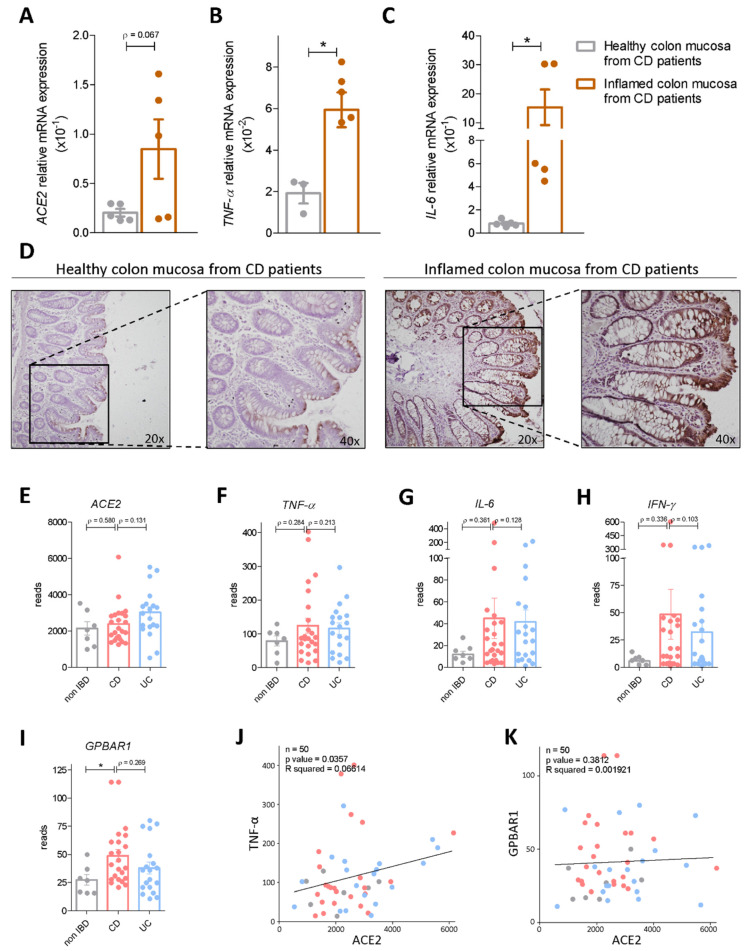
ACE2 mRNA expression in healthy and inflamed colon. The relative mRNA expression of (**A**) ACE2 (**B**) TNF-A (**C**) IL-6 in colon biopsies from healthy and inflamed colon mucosa from Crohn’s disease (**C**,**D**) patients. Data are normalized to GAPDH mRNA. Results are the mean ± SEM of 5 patients per group; * *p* <0.05. (**D**) Immunohistochemistry analysis of ACE2 expression in non-inflamed and inflamed colon samples obtained from CD patients (magnification 20× and 40×). (**E**–**I**) RNA-seq analysis of colonic biopsy samples from HMP2 project CD, UC and non-IBD patients. Each dot represents a patient. Gene profile expression of (**E**) ACE2, (**F**) TNF-A, (**G**) IL-6, (**H**) IFN-γ and (**I**) GPBAR1. (**J**,**K**) Correlation graphs between (**J**) TNF-α and ACE2 expression and (**K**) GPBAR1 and ACE2. * *p* < 0.05.

First we investigated the expression levels of ACE2 mRNA in biopsy samples obtained from ileal and colon specimens from seven CD patients who underwent terminal ileum and right colon resection for CD. Biopsy samples were collected from both macroscopically inflamed and non-inflamed post-surgical specimens. qPCR analysis demonstrated that ileal and colon expression of ACE2 mRNA is oppositely regulated in response to inflammation in CD patients (Figure 1 and Figure 2). Thus, while the expression of two inflammatory biomarkers (TNF-α and IL-6) increased significantly in both tissues (Figure 1B,C and Figure 2B,C), the ileal expression of ACE2 decreased dramatically in the inflamed ileum (Figure 1A) but increased in the right colon (Figure 2A). The opposite pattern of ACE2 expression in response to inflammation between the ileum and colon was also confirmed by immunohistochemical analysis with anti-ACE2 antibody (Figure 1D and Figure 2D). To confirm these findings in a larger population we assessed the expression of ACE2 mRNA from CD, UC and non-IBD patients enrolled in the HMP2 project [30]. The analysis of the HMP2 cohort confirmed a robust reduction in ileal expression of ACE2 mRNA in CD patients in comparison to non-IBD patients while no changes were observed in the ileum of UC patients (Figure 1E). Further analysis of the expression of inflammatory biomarkers demonstrated a strong negative correlation between the expression of IFN-γ mRNA and expression of ACE2 mRNA (Figure 1F–H,J; n = 66, *p* value < 0.0001, R squared = 0.278), strongly confirming previous data and demonstrating that ACE2 mRNA in the ileum is negatively regulated in response to inflammation by IFN-γ. In contrast to the ileum, we found that the colonic expression of ACE2 mRNA was increased in both CD and UC, in comparison to non-IBD samples, and that these changes were mirrored by similar trends in the expression of inflammatory mediators, TNF-α, IL-6 and IFN-γ (Figure 2E–H). Additionally, a positive correlation was detected among the expression of ACE2 and TNF-α mRNA in the colon (n = 50; *p* value = 0.0357; R squared = 0.06614), indicating that in contrast to the ileum, TNF-α functions as a positive regulator of ACE2 expression in the colon, (Figure 2J) as already demonstrated in previous studies by other groups [14].

### 3.2. Interaction of Bile Acid Signaling and ACE2 in Ileum and Colon

Because the above mentioned data illustrate that inflammation functions as a negative modulator of ileal ACE2 mRNA but increases colonic ACE2 mRNA and protein in IBD patients, we have investigated whether other factors might be regulated in a similar manner and found that expression of GPBAR1, a bile acid activated receptor, is robustly increased in the ileum of CD patients and that its expression is negatively correlated with the expression of ACE2 mRNA, suggesting that the G protein-coupled receptor might function as a negative regulator of ACE2 mRNA (n = 66; *p* value < 0.001, R squared = 0.167) (Figure 1I,K). However, a different situation was observed in the colon, since, while no correlation was detected among the colon expression of GPBAR1 and ACE2 in IBD patients, the expression of the two genes was higher in IBD patients in comparison to non-IBD individuals and GPBAR1 gene expression was clearly upregulated in CD patients (*p* value = 0.04 vs. non IBD) (Figure 2I,K) [14,34]. Together, these data suggest that along with inflammatory cytokines, GPBAR1 might function as a negative modulator of ACE2 in the ileum but as a positive regulator of ACE2 expression in the colon.

### 3.3. Regulation of Ace2 Expression by GPBAR1 in Mouse Models of Intestinal Inflammation

Because this different pattern of regulation of ACE2 by GPBAR1 is intriguing and GPBAR1 ligands exert potent anti-inflammatory effects in rodent models of colitis, we have begun an effort to dissect GPBAR1-ACE2 signaling in rodent models of colitis and colon cells.

In a preliminary setting, we have investigated whether colon expression of ACE2 is modulated in a rodent model of IBD. To this purpose we first assessed the colon expression of Ace2 in mice administered with oxazolone or TNBS (two models of human CD) or DSS (a model of human UC) [31] and found that all three agents promoted a severe inflammatory response, as measured by strong upregulation of the expression of pro-inflammatory markers Tnf-α, Il-1β, Il-6 and Ifn-γ in the colon, and that these changes were associated with a robust increase in the expression of Ace2 mRNA, in agreement with results obtained in IBD patients, shown in Figure 2 and Figure S1). Since these findings indicate that these three mouse models of inflammation cause a similar level of regulation of ACE2 in the colon, we then investigated how GPBAR1 regulates ACE2 mRNA expression in the mouse models of colitis.

**Figure 3:**
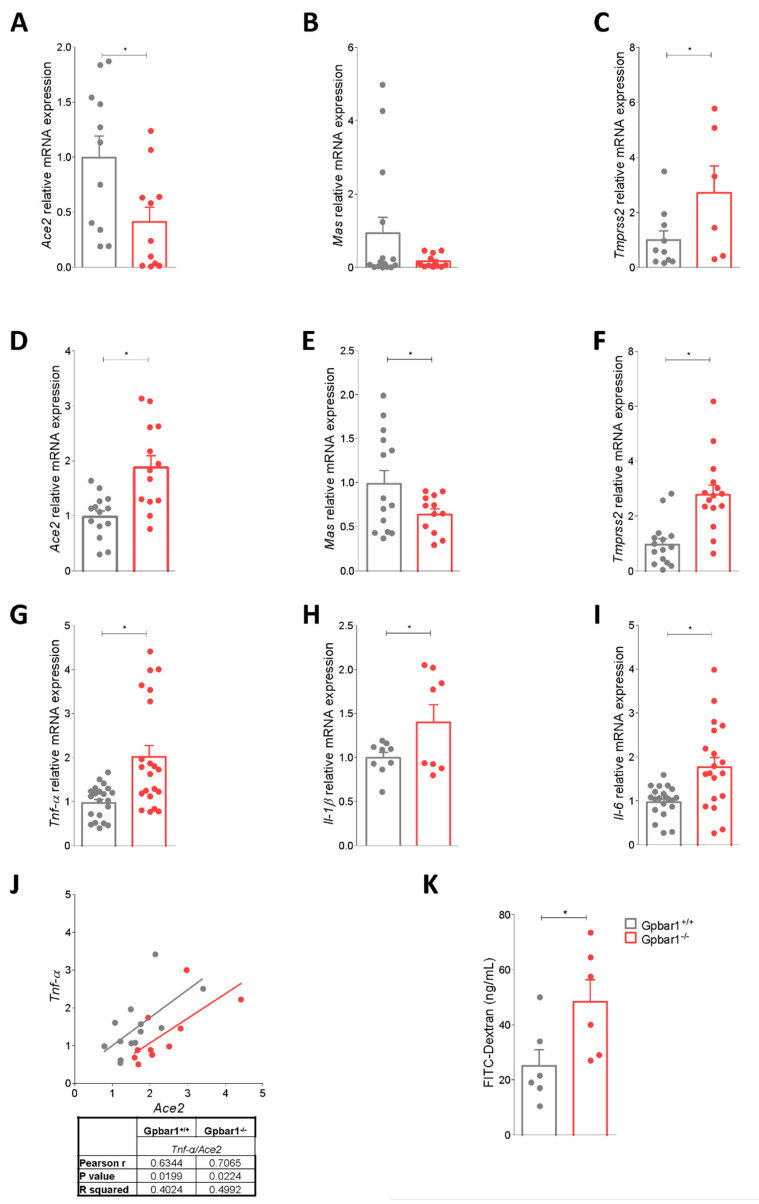
Gpbar1^−/−^ mice colon show higher levels of Ace2 expression than wild type. The Colon was collected from C57BL/6 male mice (Gpbar1^+/+^) and their congenic Gpbar1 knock out littermates (Gpbar1^−/−^). Small intestine relative mRNA expression of (**A**) Ace2, (**B**) Mas, (**C**) Tmprss2. Colon relative mRNA expression of (**D**) Ace2, (**E**) Mas, (**F**) Tmprss2 (**G**) Tnf-α, (**H**) Il-1β and (**I**) Il-6. Data are normalized to Gapdh mRNA. Results are the mean ± SEM, each point represents a mouse; * *p* < 0.05. (**J**) Correlation graph between Ace2 and Tnf-α expression. (**K**) FITC-dextran (ng/mL).

A preliminary characterization of ACE2 mRNA expression in wild type and Gpbar1^−/−^ mice demonstrated that Gpbar1 gene ablation results in a differential regulation of ACE2 expression in the small intestine and colon. Indeed, as shown in Figure 3A–C, while the ileal expression of Ace2 and Mas (an ACE2-related receptor) was markedly reduced by Gpbar1 gene ablation, the opposite took place in the colon (Figure 3D). Because we have previously shown that Gpbar1 gene ablation promotes colon inflammation [16,21,23], we examined the expression of biomarkers of colon inflammation in the two strains and found that mice harboring a disrupted Gpbar1 exhibit a robust increase in Tnf-α, IL-1β and Il-6 (Figure 3G–I) along with a marked increase in intestinal permeability (Figure 3K) with a correlation between expression levels of Tnf-α and Ace2 mRNAs (Figure 3J), suggesting that inflammation driven by Gpbar1 gene ablation prevails as a direct effect of the bile acid-activated receptor in vivo [35].

The other two genes of the Ace2 pathway, Mas and Tmprss2, show an opposite trend: the expression of Mas was downregulated in Gpbar1^−/−^ mice compared to wild-type, while Tmprss2 was upregulated (Figure 3).

**Figure 4:**
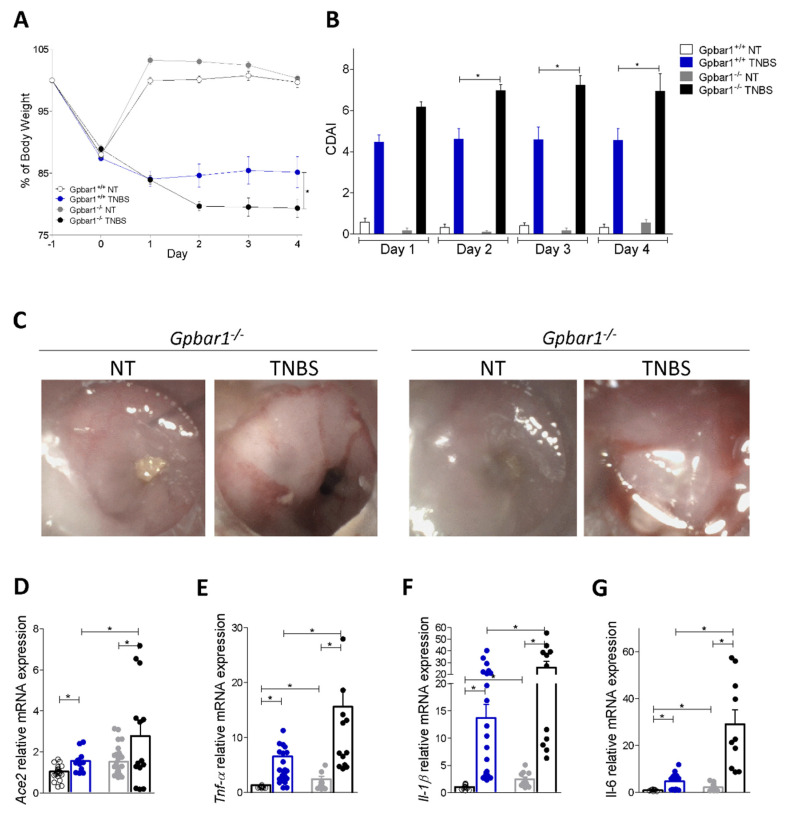
The lack of Gpbar1 increases upregulation of Ace2 in inflamed mouse colons. C57BL/6 male mice (Gpbar1^+/+^) and their congenic littermates Gpbar1 knock out (Gpbar1^−/−^) were treated with TNBS as described above. Data shown are: (**A**) changes in body weight and (**B**) Colitis Disease Activity Index (CDAI) of mice during the course of TNBS-induced colitis. (**C**) Representative endoscopic pictures showing the colon of Gpbar1^+/+^ and Gpbar1^−/−^ healthy mouse and treated with TNBS. Relative mRNA expression levels of (**D**) Ace2 and pro-inflammatory genes (**E**) Tnf-α, (**F**) Il-1β, and (**G**) Il-6. Data are normalized to Gapdh mRNA. Results are the mean ± SEM, each point represents a mouse; * *p* < 0.05.

To further dissect the role of inflammation and GPBAR1 in regulating the colon expression of ACE2, we have examined the effect of challenging wild type and Gpbar1^−/−^ mice with TNBS. As shown in Figure 4, Gpbar1^−/−^ gene ablation worsened the severity of disease, as demonstrated by assessing the body weight trend, the colitis disease activity index (CDAI) and endoscopy (Figure 4A–C). Consistent with these clinical/endoscopic features, analysis of pro-inflammatory cytokine demonstrated that while exposure to TNBS upregulated the expression of various inflammatory mediators, Tnf-α, Il-1β and Il-6 in both genotypes, these changes were robustly enhanced by Gpbar1 gene ablation (Figure 4E). Analysis of Ace2 expression confirmed a robust induction of Ace2 in response to TNBS and the fact that this regulation was exacerbated by Gpbar1 gene ablation (Figure 4D) with a direct correlation of Ace2 and Tnf-α mRNAs (data not shown) [36,37]. Together, these data reaffirmed that Ace2 expression is upregulated in the colon following the induction of colitis and that the absence of Gpbar1 enhances this mechanism.

### 3.4. BAR501, a Selective GPBAR1 Agonist Regulates ACE2 Expression In Vivo

**Figure 5:**
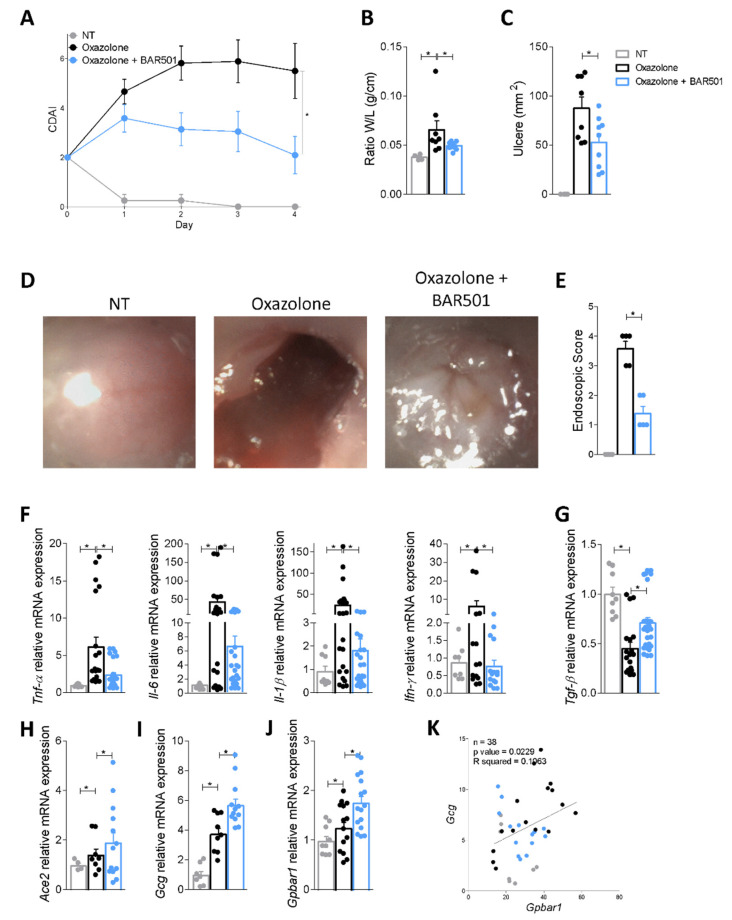
Administration of the GPBAR1 agonist further increases Ace2 expression. Colitis was induced in Balb/c male mice with administration of oxazolone per rectum alone or in combination with BAR501 (30 mg/Kg). Disease severity was scored by the following evaluations (**A**) Colitis Disease Activity Index (CDAI), (**B**) ratio of weight (W)/length (L) (g/cm) of colon, (**C**) colonic macroscopic ulcers (mm^2^), (**D**) endoscopy images and (**E**) endoscopic score. Relative mRNA expression levels of (**F**) Tnf-α, Il-1β, Il-6 and Ifn-γ; (**G**) the anti-inflammatory gene Tgf-β; (**H**) Ace2; (**I**) Gcg and (**J**) Gpbar1. Data are normalized to Gapdh mRNA. Results are the mean ± SEM, each point represents a mouse; * *p* < 0.05. (**K**) Correlation graph between Gcg and Gpbar1 expression.

Because the data support the notion that regulation of ACE2 expression in the colon is regulated by GPBAR1 but the effect of Gpbar1 gene ablation is obscured by the fact that Gpbar1^−/−^ mice develop a severe inflammation, we further dissected these signaling pathways by challenging wild type mice with colitis induced by administration of oxazolone with BAR501, a potent GPBAR1 agonist, which effectively regulates intestinal inflammation in Gpbar1-dependent manner [23]. Results shown in Figure 5 illustrate that BAR501 protects against development of signs and symptoms of colitis caused by oxazolone, as shown by assessing the CDAI, the colonic macroscopic features, endoscopy (Figure 5A–E) and colon expression of pro-inflammatory cytokine and Tgf-β (Figure 5F,G), while upregulating the expression of Ace2 by approximately 30% compared to the level of expression measured in mice treated with oxazolone alone (Figure 5H). Because these findings demonstrate that the direct Gpbar1 agonist functions as positive regulator of Ace2 expression in vivo and that this effect is maintained independently from the level of inflammation, we examined whether a correlation exists between the levels of expression of Ace2 and Gpbar1 and its target gene, GLP-1, in the colons of mice treated with BAR501. Since activation of GPBAR1 promotes the synthesis and release of GLP-1 from intestinal L cells [38], while GLP-1 regulates ACE2 expression in the heart [29], we speculated that a GPBAR1–GLP-1 axis could be involved in the regulation of ACE2 expression in response to direct activation of GPBAR1 by BAR501. The results of these assays demonstrate that BAR501 significantly increases the expression of Gcg, the gene encoding for GLP-1 in mice, and that a direct correlation exists between Gpbar1 and Gcg expression in the colon of mice with colitis induced by oxazolone (*p* value = 0.0229, Figure 5I–K). To confirm these findings, we reassessed the expression of GPBAR1 and GCG in the HMP2 cohort and found a direct correlation between the colon expression of GPBAR1 and GCG mRNAs (*p* = 0.0219) in these human samples as well (Figure S2).

**Figure 6:**
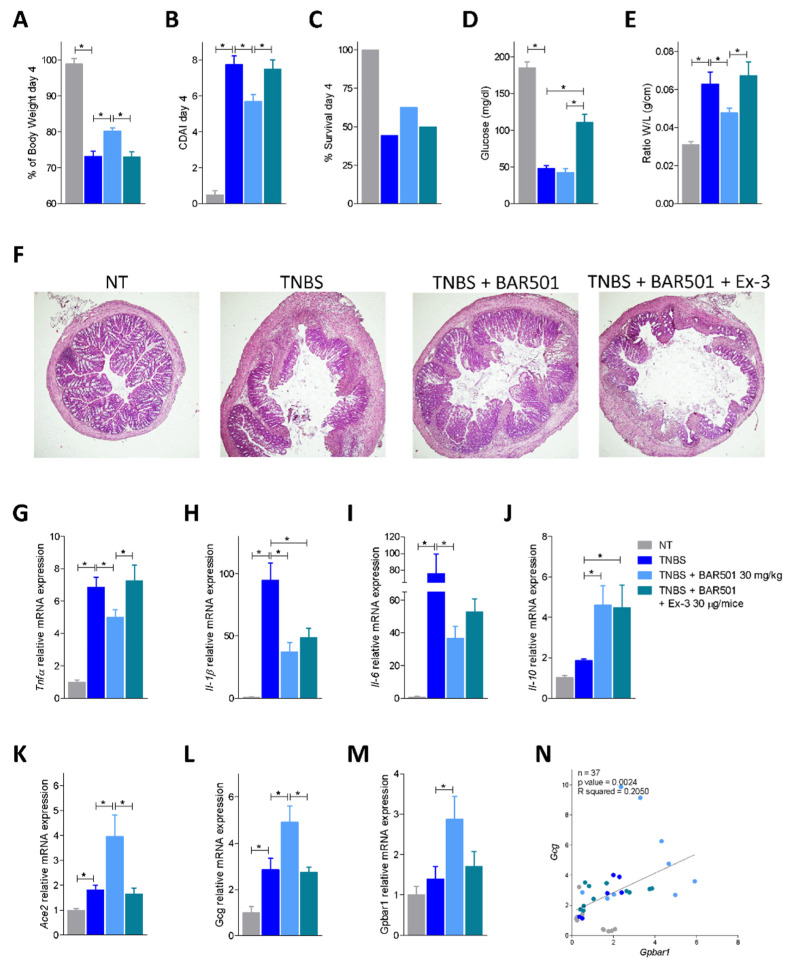
Bar501-dependent Ace2 upregulation effect was suppressed by co-administration of Bar501 and Ex-3 in a Gpbar1/Gpl-1/Glp-1R axis-dependent manner. Bar501 (30 mg/Kg) or a combination of BAR501 plus exendin-3 (Ex-3) (30 μg/mice), a GLP-1R antagonist, were administrated in C57BL/6J male mice on day 0 of a colitis TNBS model, for an additional 3 days. The fourth day was the sacrifice day. Data shown are: (**A**) trends of body weight on day 4, in percentage; (**B**) Colitis Disease Activity Index (CDAI) of mice on day 4; (**C**) percentage of survival on day 4; (**D**) glucose levels (mg/dL) in response to OGTT; (**E**) ratio of weight (W)/length (L) (g/cm) of colon; (**F**) hematoxylin and eosin (H&E) staining of colon (magnification 4×). Total RNA extracted from collected colon was used to assess by qPCR the relative mRNA expression of pro-inflammatory genes: (**G**) Tnf-α, (**H**) Il-1β and (**I**) Il-6; the anti-inflammatory gene (**J**) Il-10; (**K**) Ace2 (**L**) Gcg and (**M**) Gpbar1. Data are normalized to GAPDH mRNA. Results are the mean ± SEM, each point represents a mouse; * *p* < 0.05. (**N**) Correlation graph between Gcg and Gpbar1 expression.

Because these data strongly highlight a direct correlation between GPBAR1 and GLP-1 expression and GLP-1 and ACE2, we then investigated whether blocking GLP-1 signaling in the TNBS model of colitis reverts upregulation of ACE2 in response to BAR501. To this end, mice with colitis induced by exposure to TNBS were administered with BAR501, 30 mg/kg orally, alone or in combination with exendin-3, a GLP-1R antagonist (Figure 6). The results of these experiments demonstrated that while BAR501 reverts the inflammation and upregulates the expression of Ace2 mRNA, these beneficial effects were completed reversed by exendin-3 (Figure 6A–F). Administration of the GLP-1R antagonist also abrogated the beneficial effects of BAR501 on biomarkers of inflammation, demonstrating that the GLP-1/GLP-1R axis is involved in the protective effects exerted by the GPBAR1 agonist (Figure 6G–I). Further confirming this view, we found that the expression of Gcg and Gpbar1 mRNA exhibit the same pattern of regulation of Ace2 mRNA (Figure 6L,M) and a correlation study showed a direct correlation between the expression of Gcg and Gpbar1 (*p* = 0.0024, Figure 6N). Finally, exendin-3 exerted no effect on the upregulation of Il-10 induced by BAR501, that, as already shown [23], is directly mediated by a GPBAR1/PKA/pCREB axis (Figure 6J).

### 3.5. Effect of Activation of GPBAR1 on the Colorectal Adenocarcinoma HT29 Cell Line

Because the immunohistochemistry data shown in Figure 2D demonstrate that in the colon ACE2 is mostly expressed in epithelial cells, we then investigated how ACE2 is regulated in response to GPBAR1 activation in colon epithelial cells. For this purpose, we used HT29 cells, a human adenocarcinoma cell line. These cells have been widely used as an in vitro model for colon epithelial cells and are known for their ability to respond to inflammation induced by bacterial endotoxin (LPS) and TNF-α in a nuclear factor (NF)-kB-dependent manner [39].

**Figure 7:**
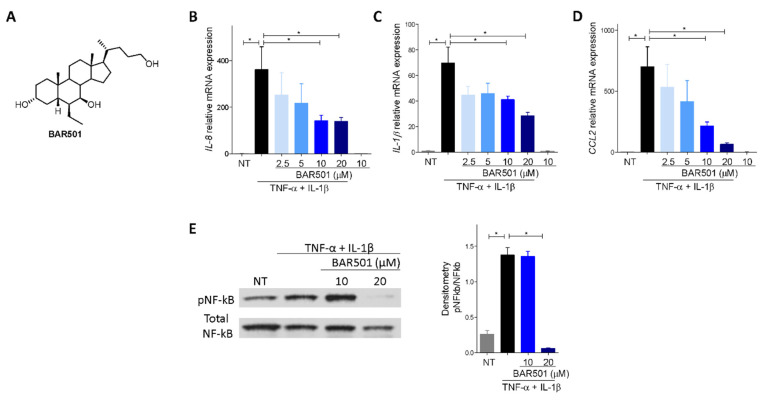
Effect of activation of GPBAR1 on the colorectal adenocarcinoma HT29 cell line. (**A**) Chemical structure of BAR501, a selective agonist of GPBAR1. (**B**) Quantitative real-time PCR analysis of cytokines (**B**) Il-8 and (**C**) Il-1β and chemokines (**D**) Ccl2 in Ht29 cell line activated with TNF-α 100 ng/mL an IL-1β 25 ng/mL for 24 h alone or in combination with BAR501 at different concentration (2.5, 5, 10, 20 µM). The data are normalized to GAPDH mRNA. Results are expressed as mean ± SEM; * *p* < 0.05. (**E**) Western blot analysis of p-NF-kb levels in HT29 cells in presence of BAR501 at 10 and 20 µM and densitometric analysis performed with Image J from blots corresponding to all samples, data are presented as mean ± SE relative to total NF-kb levels.

**Figure 8:**
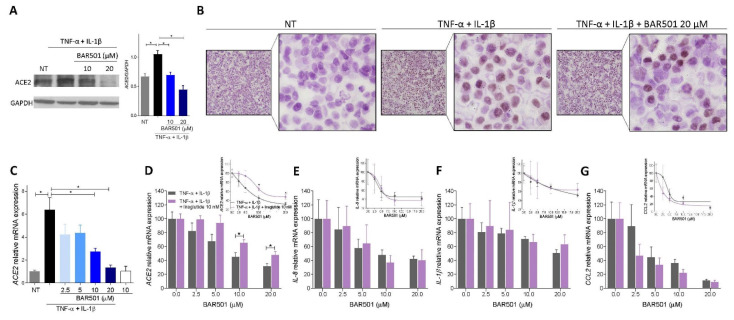
Liraglutide potentiates the anti-inflammatory activity of GPBAR1 in vitro. (**A**) Western blot analysis of Ace2 expression in HT29 cells in presence of BAR501 at 10 and 20 µM and densitometric analysis performed with Image J from blots corresponding to all samples, data are presented as mean ± SE relative to GAPDH. (**B**) Immunocitochemistry with anti-ACE2 antibody on HT29 cells (Magnification 4× and 10×). (**C**) Quantitative real-time PCR analysis of Ace2 expression in Ht29 cell line activated with TNF-α 100 ng/mL an IL-1β 25 ng/mL for 24 h alone or in combination with BAR501 at different concentration (2.5, 5, 10, 20 µM). Relative mRNA expression of (**D**) Ace2, (**E**) Il-8, (**F**) IL-1 β and (**G**) Ccl2 in HT29 cell line exposed to different conditions (TNF-α 100 ng/mL an IL-1β 25 ng/mL for 24 h alone or in combination with liraglutide (10 nM) and BAR501 at increasing concentrations (2.5, 5, 10, 20 µM) evaluated by real-time PCR. The data are normalized to GAPDH mRNA. Results are expressed as mean ± SEM; * *p* < 0.05.

As shown in Figure 7, exposure of HT29 to TNF-α in combination with IL-1β, resulted in NF-KB phosphorylation, leading to robust induction of IL-8, IL-1β and CCL2 gene transcription (Figure 7B–E). This effect was reversed by co-treating HT29 cells with BAR501, a selective GPBAR1 agonist [40] (Figure 7A), in a concentration-dependent manner. At the concentration of 20 µM, BAR501 completely reversed NF-kB phosphorylation and robustly attenuated transcription of IL-8, IL-1β and CCL2 (Figure 6B–F) [35]. Consistent with in vivo findings, exposure of HT29 cells to inflammation resulted in a significant upregulation of ACE2 mRNA (≈8 times) and protein (≈1.5 times) (Figure 8A–D). This effect was reversed in a concentration dependent manner by co-treating the cells with BAR501, strongly demonstrating that ACE2 gene expression in the colon epithelial cells is NF-kB dependent and is induced in response to inflammation [14,34,41,42].

Because the in vivo results demonstrate that regulation of Ace2 mRNA expression in response to BAR501 in the TNBS model of colitis involves a GLP-1-dependent mechanism, we then investigated whether liraglutide, a GLP-1 receptor agonist, reverses the inhibitory effects exerted by the GPBAR1 agonist on ACE2 expression in vitro. Results shown in Figure 8D–G, demonstrated that adding liraglutide to the incubation mixture produced a shift to the right of the concentration–response curve of the effects exerted by BAR501 and partially reduced the downregulation exerted by the GPBAR1 agonist on ACE2 mRNA (Figure 8D), while it did not interfere with the anti-inflammatory effects exerted by BAR501 as measured by assessing the expression levels of IL-8, IL-1β and CCL2 mRNA (Figure 8E–G).

**Figure 9:**
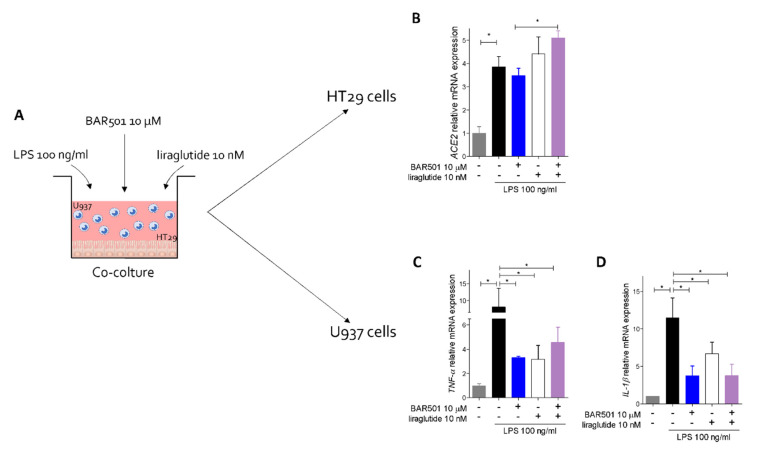
Regulation of ACE2 and inflammation by GPBAR1 agonism in colon epithelial cells and macrophages co-coltures. (**A**) HT29 and U937 cells were used to perform a coculture experiment, as described in Materials and Methods Section. (**B**) Relative mRNA expression levels of ACE2 gene in HT29 cells; relative mRNA expression levels of cytokines (**C**) IL-1β and (**D**) TNF-α in U937 cells. The data are normalized to GAPDH mRNA. Results are expressed as mean ± SEM; * *p* < 0.05.

Additionally, as shown in Figure 9, while exposing HT29 and U937 cells, a human macrophage cell line, co-cultures to lipolysaccharide (LPS) increased the expression of ACE2 in HT29 cells along with TNFα and IL-1β in U937 cells, these effects were differentially modulated by GPBAR1 agonism. Thus, while BAR501 greatly attenuated cytokine production from U937 cells, the GPBAR1 agonist exerted no effect on ACE2 mRNA expression in HT29 cells. However, adding liraglutide to the medium, significantly increased ACE2 mRNA expression in HT29 cells (Figure 9B–D), confirming that while GPBAR1 functions as a negative modulator of TNF-α release from immune cells, it also promotes ACE2 expression by GLP-1-mediated mechanisms.

## 4. Discussion

In this study we report that the bile acid receptor GPBAR1 functions a positive modulator of ACE2 expression in the colon by a mechanism that involves the release of GLP-1. ACE2 has attracted wide attention since the discovery that this carboxypeptidase, whose main function is to generate Ang-(1-7) from Ang II, functions as the entry point for SARS-CoV-2 into human cells [43]. Because mapping of ACE2 expression has revealed that the small intestine and colon are among the human tissues that host higher expression of the receptor, regulation of ACE2 expression in the gastrointestinal tract might provide a therapeutic opportunity [34,44].

The functional role of ACE2 in the gastrointestinal tract remains poorly characterized. In the small intestine, ACE2 is coexpressed with B^0^AT1, a sodium-dependent neutral amino acid transporter. The two proteins are obligatory partners and assemble as a heterodimer that is a part of quaternary complex that putatively imports amino acids, such as tryptophan, into enterocytes [45]. The functional role of ACE2 in the colon is less characterized although the receptor undergoes a robust regulation in response to inflammation [46].

In the present study we have confirmed that transcription of ileal and colon ACE2 undergoes a site-specific regulation in response to inflammation. Using, a local cohort of Crohn’s disease patients we demonstrate that, while ileal ACE2 mRNA is downregulated in Crohn’s disease patients, the colon expression of the receptor is increased. These findings were validated by an external cohort of IBD patients enrolled in the Human Microbiome Project, part 2 (HMP2) [30]. In both cohorts, we found that the expression of ileal ACE mRNA is reduced in Crohn’s disease in comparison with healthy controls and that ileal ACE2 mRNA expression negatively correlates with the tissue expression of IFN-γ. In contrast, in the colon, the expression of ACE2 mRNA increases in response to inflammation, showing a direct correlation with the tissue expression of TNF-α. Our findings are therefore consistent with previous reports showing that ileal and colon ACE2 mRNAs are differentially regulated in response to inflammation in IBD patients [14,34,41,42].

Since intestinal inflammation differentially modulates ileal and colon expression of ACE2 mRNA in Crohn’s disease patients, we further investigated the mechanisms involved in the regional regulation of ACE2 and focused our attention on the bile acid receptor GPBAR1. GPBAR1 is a bile acid receptor for secondary bile acids, LCA and DCA, that are formed in the colon from enzymatic processing of primary bile acids, CA and CDCA, by the intestinal microbiota [10,11,12], We have previously shown that, in addition to exerting potent counter-regulatory effects on intestinal inflammation, and attenuating inflammation-driven immune dysfunction in rodent models of IBD [17,23,47,48,49], bile acids might bind and activate ACE2 [50], suggesting a potential interaction between these pathways. Additionally, since GPBAR1 agonism attenuates TNF-α production by M1 type intestinal macrophages and promotes IL-10 mRNA transcription, we decided to investigate how GPBAR1 expression is regulated in the terminal ileum and colon of IBD patients and whether this pattern of regulation is integrated with ACE2 mRNA expression. The results of these analyses, shown in Figure 1 and Figure 2, demonstrated that in contrast to ACE2, GPBAR1 gene expression was significantly upregulated both in the ileum and colon of Crohn’s disease patients and that a negative correlation exists between GPBAR1 gene expression and ACE2 in the terminal ileum, but not in the colon. These regional differences, however, suggest that reciprocal regulation of GPBAR1 and ACE2 gene expression is indirect and additional factors are involved.

To further dissect these pathways, we decided to investigate the phenotypic expression of ACE2 mRNA in Gpbar1^−/−^ mice. We and others have reported that Gpbar1 gene ablation results in a pro-inflammatory intestinal phenotype, marked by a robust upregulation of biomarkers of inflammation, including Il-1β, Tnf-α, and increased intestinal permeability along with a disrupted maturation of intestinal tight junctions [23,27]. The mapping of Ace2 mRNA in naïve Gpbar1^−/−^ mice revealed, that expression of ileal and colon ACE2 was robustly regulated in these mice in comparison to their congenic littermates. Thus, while Ace2 mRNA was markedly reduced in the ileum, the opposite took place in the colon. In contrast, two downstream genes in the ACE2 pathway, Mas and Tmprss2, followed an independent pathway of regulation. Indeed, while Mas expression was robustly reduced in the ileum and colon of Gpbar1^−/−^ mice in comparison to naïve wild type mice, the expression of Tmprss2 mRNA was upregulated in both tissues (Figure 1 and Figure 2). This pattern of regulation therefore suggests that tissue inflammation rather than GPBAR1 gene expression modulates ACE2 expression.

We focused our attention on how colon ACE is regulated in response to colitis in rodent models of IBD and, as illustrated in Figure S1, we found that Ace2 mRNA was robustly upregulated in three models of colitis induced by administration of oxazolone, TNBS and DSS, confirming previous animal [35] and human data [14,34,41,42]. Furthermore, confirming the fact that GPBAR1 functions as a braking mechanism for intestinal inflammation [23], mice harboring a disrupted Gpbar1 develop a progressive inflammation with age and were more prone to develop severe disease than their wild type counterparts when challenged with TNBS, as shown by assessing the colitis activity score (CDAI) and expression of Tnf-α and Il-1β mRNA, and this pattern associated with a very robust induction of Ace2 mRNA expression in the colon. As in Crohn’s disease patients, colon expression of Ace2 in TNBS mice demonstrated a linear correlation with the expression of inflammatory genes, specifically Tnf-α, indicating that this cytokine is an effective positive modulator of colon ACE2 in both human and mice.

Because the overwhelming role exerted by colon inflammation in regulating Ace2 mRNA expression in this region prevents gaining information on the role of GPBAR1 per se, we investigated whether activation of GPBAR1 in two rodent models of colitis directly modulates ACE2 mRNA expression. The results of this study demonstrated that treating mice rendered colitic by administration of oxazolone and DSS with BAR501, a GPBAR1 agonist, protected against the development of colitis, attenuated the signs and symptoms of colitis and reversed the immune dysfunction that typically occurs in this model, although upregulated the expression of Ace2 in the colon. These data demonstrate that, in the colon, GPBAR1 functions as a positive regulator of ACE2, which is consistent with the general view that ACE2 is an anti-inflammatory gene, as demonstrated by the fact that Ace2 gene ablation worsens the severity of inflammation in mouse models of colitis and angiotensins 1–7 exert direct anti-inflammatory effects [37]. Together, these data, suggest that regulation of ACE2 mRNA in the setting of colon inflammation might contribute to the counter-inflammatory effects exerted by GPBAR1 [15].

Further examining the transcription profile of GPBAR1 target genes in these models, we found that BAR501 markedly induced the expression of its target genes GLP-1 [24,26], and a correlation analysis carried out on human samples confirmed that expression GPBAR1 was directly correlated with expression of GCG (the gene encoding for human GLP-1) [51]. Additionally, we detected a trend for a positive correlation, thought it was not significant, between the expression of GCG and ACE2 mRNAs both in human samples of Crohn’s disease patients and DSS mice. Because GLP-1 modulates ACE2 expression in the cardiovascular system [29], we performed a series of studies to further elucidate the potential role of GLP-1 in regulating colon ACE2 expression in response to GPBAR1 agonism, and found that both the anti-inflammatory effects exerted by BAR501 in the TNBS model of colitis and positive regulation of Ace2 mRNA in the colon of these animals, were reversed by co-treating mice with exendin-3, a GLP-1-R antagonist [52]. Again, in this setting, colon expression of Ace2 mRNA was dissociated from inflammation, since, despite the fact exendin-3 worsened the severity of inflammation and immune dysfunction, the GLP-1-R antagonist dramatically downregulated the expression of Ace2 mRNA, as well as the expression of Gcg and Gpbar1 (Figure 6). Taken together, these data suggest that while Ace2 mRNA is induced in response to inflammation, a specific regulatory mechanism may supersede this regulation, and that activation of the GPBAR1/GLP-1/GLP-1-R axis effectively modulates the expression of Ace2 independently from inflammation.

We further dissected the molecular mechanisms involved in ACE2 regulation by GPBAR1 by in vitro studies using HT19 cells, a colon epithelial cell line. Results from these studies provided evidence that expression of ACE2 mRNA is directly induced by exposure to TNF-α and IL-1β and that inhibition of NF-kB phosphorylation by the GPBAR1 agonist BAR501, abrogates both the pro-inflammatory response and the positive regulation of ACE2 mRNA in these cells. However, co-treating the cells with liraglutide, a GLP-1 analogue, and exendin-4, a GLP-1R agonist [52], reinduced the expression of ACE2 mRNA without interfering with the anti-inflammatory effect of BAR501. This finding was confirmed by results obtained in vitro using colon epithelial cells and macrophages co-cultures. In this system, similar to the in vivo findings, GPBAR1 agonism blunted the inflammatory response caused by LPS in macrophages but was less effective in modulating ACE2 mRNA in colon epithelial cells, and addition of liraglutide strongly promoted ACE2 mRNA expression.

In addition to GLP-1, our in vivo experiments have shown that BAR501 functions as a positive modulator of IL-10 [23]. Because IL-10 is a potent inducer of ACE2 in endothelial cells [53], we cannot rule out the fact that in vivo, this cytokine might contribute to the regulatory effect BAR501 exerts on Ace2 mRNA in the whole colon.

On the other hand, while ACE2 mRNA expression in the colon is induced by inflammation, and while enhanced expression of colon ACE2 mRNA does not reduce the efficacy of pharmacological treatments in IBD patients [42], the fact that CD patients harboring a higher expression of ACE2 mRNA seem to develop a more aggressive disease [41] and are at higher risk for surgery [41], suggests that ACE2 could be considered a potential biomarker of inflammation and immune dysfunction in IBD.

In summary, we have shown that the bile acid receptor GPBAR1 regulates the colon expression of ACE2 by a GLP-1-dependent mechanism. These data highlight that modulation of the GPBAR1/GLP-1/ACE2 axis might be beneficial in treating IBD.

## Data Availability

Data supporting reported results can be found in GSE111889 series that includes gene expression profiles (RNA-seq analysis, Illumina HiSeq 2000) of biopsy samples retrieved from CD, UC and non-IBD patients enrolled in the Human Microbiome Project, part 2 (HMP2) project (https://www.ncbi.nlm.nih.gov/geo/query/acc.cgi?acc=GSE111889 accessed on 3 February 2022).

## Supplementary Materials

The following supporting information can be downloaded at: https://www.mdpi.com/article/10.3390/cells11071187/s1, Supplementary Figure S1. The colonic Ace2 mRNA expression is upregulated in IBD and models. Colitis was induced in Balb/c male mice with administration of (A–E) Oxazolone, (F–J) TNBS and (K–O) DSS as described in Materials and Methods section. Total RNA extracted from colon was used to evaluate by quantitative real-time PCR the relative mRNA expression of (A,F,K) Ace2, (B,G,L) Tnf-α, (C,H,M) Il-1β, (D,I,N) Il-6, (E,J,O) Ifn-γ. Data are normalized to Gapdh mRNA. Results are the mean ± SEM of 5–7 mice per group; * *p* < 0.05. Supplementary Figure S2. Direct correlation between CGC and GPBAR1 expression in human colon samples. The data have been extrapolated from RNA-seq analysis of colonic biopsy samples from HMP2 project CD, UC and non-IBD patients. Each dot represents a patient. The graph represents a correlation analysis between GCG and GPBAR1 expression.
