# Supplementary figures and images for: GLP-1 Mediates Regulation of Colonic ACE2 Expression by the Bile Acid Receptor GPBAR1 in Inflammation
Source: Cells. 2022 Apr 1;11(7):1187. doi: 10.3390/cells11071187 (PMC8998127; doi:10.3390/cells11071187)

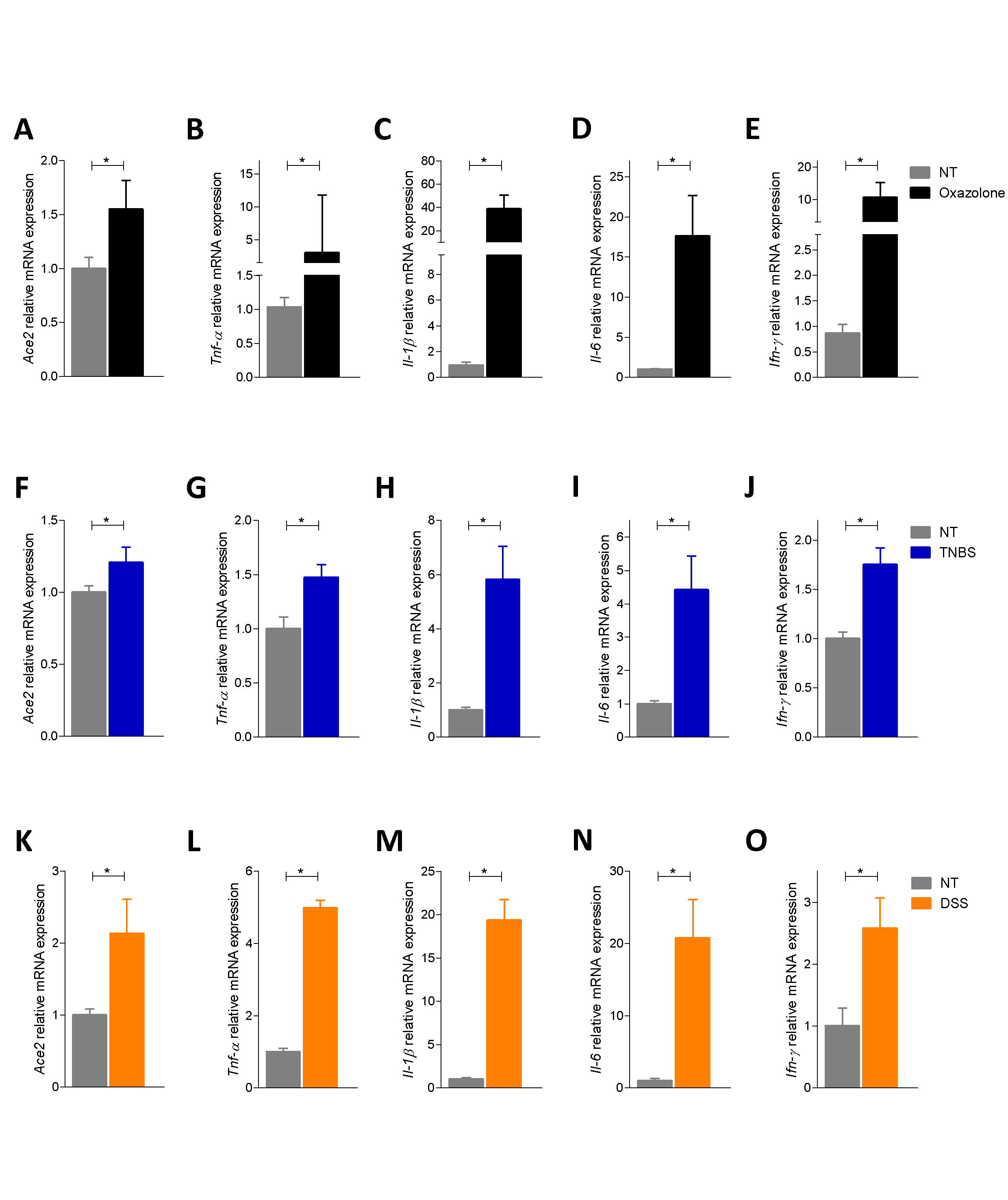

Supplement: Supplementary file 1 [file cells-11-01187-s001.zip › Figure S1.tif]

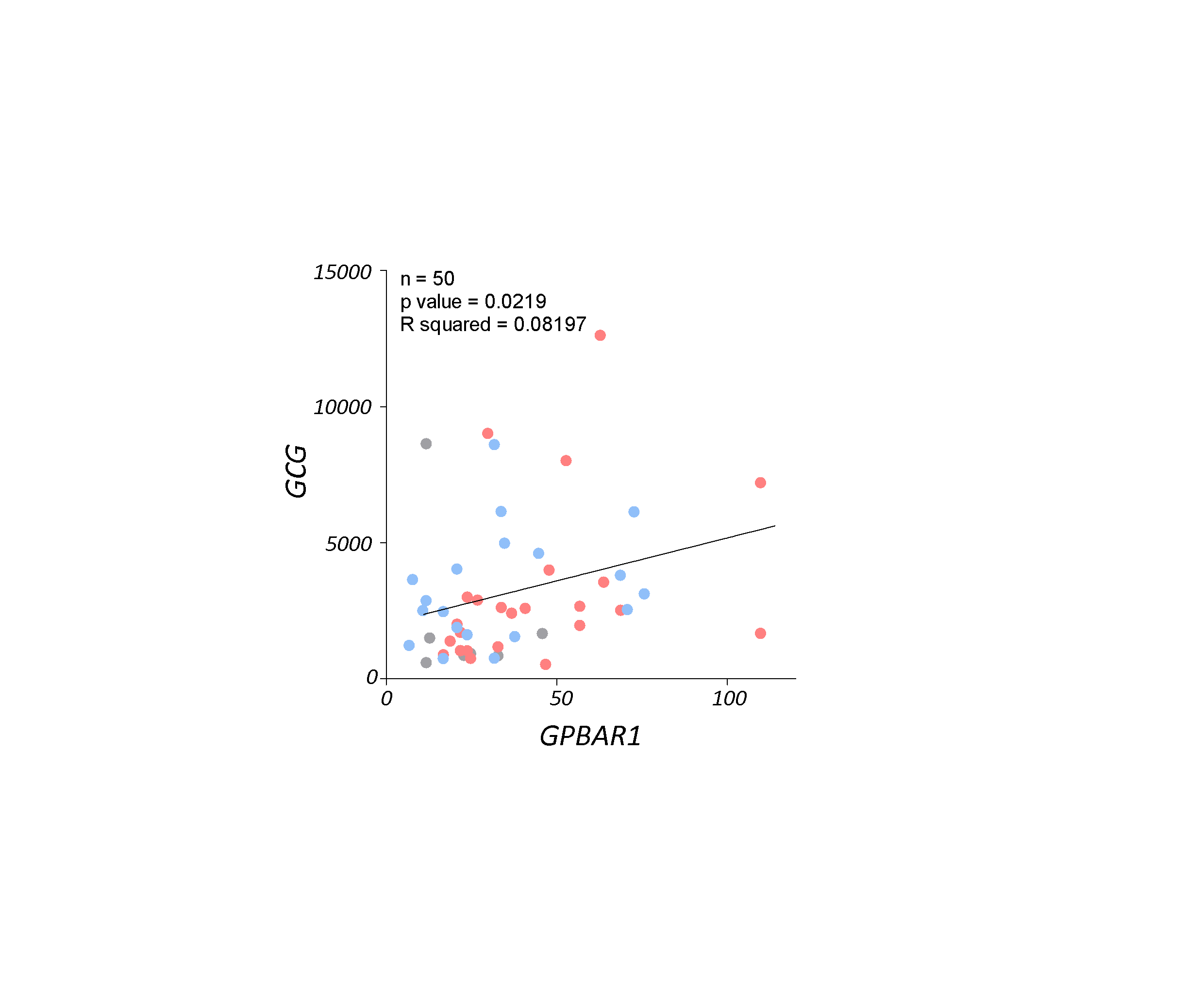

Supplement: Supplementary file 1 [file cells-11-01187-s001.zip › Figure S2.tif]
